# Value of fractional-order calculus (FROC) model diffusion-weighted imaging combined with simultaneous multi-slice (SMS) acceleration technology for evaluating benign and malignant breast lesions

**DOI:** 10.1186/s12880-024-01368-4

**Published:** 2024-07-29

**Authors:** Fei Wang, Yi-Nan Sun, Bao-Ti Zhang, Qing Yang, An-Dong He, Wang-Yan Xu, Jun Liu, Meng-Xiao Liu, Xiao-Hu Li, Yong-Qiang Yu, Juan Zhu

**Affiliations:** 1 Department of Radiology, The First Affiliated Hospital of Anhui Medical University, No.218, Jixi Road, Hefei, 230032 China; 2 Department of Radiology, Anqing Municipal Hospital, No.352, Renmin Road, Anqing, 246003 China; 3 MR Research & Marketing Department, Siemens Healthineers Co., Ltd, No.278, Zhouzugong Road, Shanghai, 201318 China

**Keywords:** Breast cancer, Fractional calculus model, Simultaneous multi-slice, Diffusion-weighted imaging, Magnetic resonance imaging

## Abstract

**Background:**

This study explores the diagnostic value of combining fractional-order calculus (FROC) diffusion-weighted model with simultaneous multi-slice (SMS) acceleration technology in distinguishing benign and malignant breast lesions.

**Methods:**

178 lesions (73 benign, 105 malignant) underwent magnetic resonance imaging with diffusion-weighted imaging using multiple b-values (14 b-values, highest 3000 s/mm^2^). Independent samples t-test or Mann-Whitney U test compared image quality scores, FROC model parameters (D,, ), and ADC values between two groups. Multivariate logistic regression analysis identified independent variables and constructed nomograms. Model discrimination ability was assessed with receiver operating characteristic (ROC) curve and calibration chart. Spearman correlation analysis and Bland-Altman plot evaluated parameter correlation and consistency.

**Results:**

Malignant lesions exhibited lower D, and ADC values than benign lesions (*P* < 0.05), with higher values (*P* < 0.05). In SSEPI-DWI and SMS-SSEPI-DWI sequences, the AUC and diagnostic accuracy of D value are maximal, with D value demonstrating the highest diagnostic sensitivity, while value exhibits the highest specificity. The D and combined model had the highest AUC and accuracy. D and ADC values showed high correlation between sequences, and moderate. Bland-Altman plot demonstrated unbiased parameter values.

**Conclusion:**

SMS-SSEPI-DWI FROC model provides good image quality and lesion characteristic values within an acceptable time. It shows consistent diagnostic performance compared to SSEPI-DWI, particularly in D and values, and significantly reduces scanning time.

## Background

Breast cancer is one of the most common malignancies in women [1], and magnetic resonance imaging (MRI) plays a key role in its diagnosis and assessment. Diffusion-weighted imaging (DWI), a conventional functional MRI method, can be combined with dynamic contrast-enhanced imaging to enhance the characterization of breast lesions, thereby reducing unnecessary biopsies of benign lesions [2]. Although apparent diffusion coefficients (ADCs) derived from the single-exponential DWI model substantially aid in distinguishing between benign and malignant breast lesions [3], water molecule diffusion within complex cell structures does not adhere to a Gaussian distribution. Therefore, a multitude of non-Gaussian distribution diffusion models have been developed, including Intravoxel Incoherent Motion (IVIM) [2], Neurite Orientation Dispersion and Density Imaging (NODDI) [4], Diffusion Kurtosis Imaging (DKI) [5], the Stretched Exponential (SEM) model [6], the Fractional Order Calculus (FROC) model [7], and the Continuous-Time Random Walk (CTRW) model [8].

Among these models, IVIM and DKI have been extensively and maturely applied in the study of breast lesions. The empirical parameter α in the SEM model presents a certain challenge to the accuracy of the model. The FROC model is similar to the CTRW model in that they both acknowledge the inhomogeneity of intra-voxel diffusion in both space and time, and the FROC model is a simplification of the CTRW model [9], and its clinical application is more extensive. Fractional-order calculus (FROC) model, which has gained attention in recent studies of non-Gaussian distributed diffusion methods, introduces new parameters such as the anomalous diffusion coefficient (D), intravoxel diffusion heterogeneity ($\:\beta\:$), and spatial constant ($\:\mu\:$) [10, 11]. Recent studies have shown that compared with single-exponential DWI and diffusion kurtosis imaging, the FROC model more effectively detects microstructural changes and heterogeneity in tumor tissues, thereby improving assessments of tissue complexity [11]. Its efficacy has been demonstrated in various cancers such as brain tumors [12, 13], cervical cancer [14], bladder cancer [15], prostate cancer [11], lung cancer [16], and liver cancer [17, 18]; however, there have been few reports concerning its use in evaluating breast lesions [19, 20]. A prior investigation using FROC-DWI with maximum b-value (b = 1500 s/mm²) and only 4 b-values indicated that ADCs were superior to FROC-DWI-derived parameters in terms of diagnostic performance [19]. Wang et al. [20] concluded that D and $\:\mu\:$ values from FROC-DWI showed promising diagnostic efficacy in distinguishing between benign and malignant breast lesions. The variability in b-values and the highest b-value in the FROC-DWI sequence require exploration to enable use of this sequence in the assessment of breast lesions.

The FROC-DWI sequence requires a multitude of b-values, typically more than five, with the highest b-value exceeding 2000 s/mm² [9]. Consequently, the scanning time for the FROC-DWI sequence is relatively lengthy, which limits its research and clinical applications. In recent years, a technology known as simultaneous multi-slice (SMS) for rapid signal acquisition has emerged. This technique significantly reduces MRI scanning time by exciting multiple slices at once during a single scan, thereby reducing the number of excitations required to cover the same range of slices. It achieves this by decreasing the repetition time (TR) through an acceleration factor, which is equivalent to the number of slices excited simultaneously. SMS, predominantly during single-shot echo planar imaging DWI (SSEPI-DWI), has been utilized in assessments of the brain [21–23], abdomen [24–26], and breast glands [27–30]. In the study of brain diffusion-weighted imaging (DWI) and diffusion tensor imaging (DTI), the introduction of simultaneous multi-slice (SMS) technology did not introduce bias in the signal-to-noise ratio (SNR), contrast-to-noise ratio (CNR), and ADC values when compared to conventional scanning, as confirmed by Bland-Altman analysis. Moreover, the fractional anisotropy (FA) values of the lesions were found to be 9% higher when using SMS technology compared to conventional scanning. Tar on and colleagues used two different SMS acceleration factors (2-fold and 3-fold) in pancreatic DWI scans. The results showed that 2-fold accelerated SMS DWI was comparable to conventional DWI in terms of image quality and lesion depiction, and the scanning time was reduced to one-third of the conventional DWI. However, the overall image quality of the 3-fold accelerated SMS DWI significantly declined, and the ADC values of the pancreatic body and tail were also markedly reduced. In breast imaging, SMS technology (with an acceleration factor of 2) was applied to the single-exponential model DWI and it was found that the image quality and diagnostic accuracy of the ADC values of SMS-SSEPI-DWI were comparable to conventional SSEPI-DWI, while significantly reducing the scanning time.

In recent years, although readout-segmented echo planar imaging (rs-EPI) has been developed to overcome the lower spatial resolution and geometric distortion issues associated with SSEPI-DWI sequences [28, 29], the relatively longer scanning time of rs-EPI has significantly limited its clinical application, especially when applied to diffusion models that require more and higher b-values, such as FROC or CTRW models. Sanderink and colleagues compared the application of rs-EPI and SMS-SSEPI DWI in 25 female patients with breast lesions [28]. Although the overall image quality of rs-EPI was superior to SMS-SSEPI and had fewer artifacts, the depiction of lesions, especially malignant ones, was more pronounced on SMS-SSEPI images, and the ADC values were found to be lower. However, few studies have explored the use of SSEPI-DWI combined with SMS technology in FROC model processing.

Therefore, this study compared the diagnostic performances of breast SMS-SSEPI-DWI and conventional SSEPI-DWI with respect to FROC model imaging time, image quality, and derived parameters (D, $\:\beta\:$, $\:\mu\:$) for distinguishing between benign and malignant breast lesions.

## Methods

### Patients

This retrospective study protocol was approved by the hospital ethics committee [approval number: Medical Lun Shen (2023) No. 99], and written informed consent was waived.

A cohort of 256 women who underwent breast MRI scans at our institution between June 2021 and September 2023 was initially collected. Inclusion criteria were age > 18 years and the completion of two sets of multi-b-value DW images during breast MRI. Exclusion criteria were prior history of radiotherapy, chemotherapy, or surgery for breast lesions (*N* = 15); absence of surgery-based or needle biopsy-based pathological confirmation of the lesion (*N* = 22); incomplete breast MRI scan before surgery or within 2 weeks prior to needle biopsy (*N* = 8); severely poor MR image quality that substantially hindered interpretation due to artifacts (*N* = 6); and lesions with a diameter of < 5 mm (*N* = 27). Ultimately, 178 patients met the study criteria; the patient age range was 24 to 78 years (mean age, 49.52 ± 12.1 years). Based on the nature of the breast lesions, patients were categorized into a benign lesion group (*N* = 73) and a malignant lesion group (*N* = 105).

### MRI data acquisition

A 3.0-T MR scanner (MAGNETOM Skyra, Siemens Healthineers, Erlangen, Germany) equipped with a 16-channel breast coil was used for imaging. The DWI sequence included 14 b-values and was conducted in two groups: conventional single-shot echo planar imaging (SSEPI-DWI) and SSEPI-DWI combined with SMS technology (SMS-SSEPI-DWI), both DWI sequences have identical scan positioning. Due to the value demonstrated in clinical practice by the diffusion of multiple b-values across a multitude of literature, we routinely scan two sets of multi-b-value sequences in our clinical work to assist in clinical diagnosis. Except for differences in repetition time, SMS acceleration factor, and scan time, parameters were largely consistent. Specific parameters are delineated in Table 1. Other scan sequences included T2-weighted imaging fat saturation with repetition time/echo time, 5000 ms/81 ms; field of view, 340 × 340 mm^2^; matrix size, 384 × 384; slice thickness/gap, 4.0 mm/1.0 mm; flip angle, 120 degrees; and acquisition time, 2 min 35 s. Transect T1-weighted imaging dynamic contrast-enhanced MRI (1 unenhanced and 6 enhanced sequence sets) included repetition time/echo time, 450 ms/1.58 ms; field of view, 340 × 340 mm^2^; matrix size, 384 × 256; slice thickness, 1 mm; flip angle, 12 degrees; and acquisition time, 7 min 20 s.

**Table 1:** DWI sequence parameters

Parameter	SSEPI-DWI	SMS-SSEPI-DWI
Field of view (mm^2^)	360 × 227.5	
Slice thickness/gap (mm)	4/0.4	
No. of slices	30	
Voxel size (mm^3^)	0.9 × 0.9 × 4	
Bandwidth (Hz/pixel)	1644	
Fat suppression	SPAIR	
b-values [s/mm^2^]/(means)	0 (1), 50 (1), 80 (1), 100 (1), 150(1), 200 (1), 400 (1), 600 (1), 800 (1), 1000 (2), 1500 (2), 2000 (2), 2500 (3), 3000 (3)	
Diffusion mode	3-scan trace	
Diffusion scheme	Monopolar	
GRAPPA acceleration factor	2	
Acceleration mode	GRAPPA	Slice GRAPPA
Repetition time (ms)	7800	3800
Echo time (ms)	87	88
Slice acceleration factor	—	2
Acquisition time (min: s)	7:48	3:55

### Image analysis

Two radiologists (WF and ZJ, with 8 and 15 years of experience in breast MRI diagnosis, respectively) independently analyzed the images. They were blinded to the pathological findings and DWI sequence parameters. After image acquisition, two sets of original DW images were uploaded to Body DiffusionLab (BoDiLab, Chengdu ZhongYing Medical Technology Co., Ltd., Chengdu, China) in the MR Station. Utilizing post-processing software, calculations were performed to derive results for each parameter of the DWI mono-exponential model and the FROC model.

1. ADC (DWI mono-exponential model) fitting formula:

$${S}_{b}/{S}_{0}= exp(-b\times ADC)$$

ADC: apparent diffusion coefficient, ${S}_{b}$: image signal intensity at b > 0 s/mm^2^, $\:{S}_{0}$: image signal intensity at b = 0 s/mm^2^.

(2) FROC model fitting formula:

$${S}_{b}/{S}_{0}=exp\left[-D{\mu\:}^{2\left(\beta -1\right)}{\left(\gamma {G}_{d}\delta \right)}^{2\beta }\left(\varDelta -\frac{2\beta -1}{2\beta+1}\delta \right)\right]$$

$\:{S}_{b}/{S}_{0}$: same meaning as in the mono-exponential model; ${G}_{d}$, $\delta$, and $\varDelta$: amplitude, pulse width (25.66 ms), and gradient interval (30.13 ms) of the diffusion gradient, respectively [31]; D: diffusion coefficient ($\mu$m^2^/ms); $\beta :$ intravoxel diffusion heterogeneity (unitless, 0 < $\:\beta\:$ ≤ 1); and $\:\mu\:$: spatial constant ($\:\mu\:$m). The Levenberg-Marquardt nonlinear fitting algorithm was used to fit the diffusion images of 14 b-values to the FROC model on a voxel-by-voxel basis, thereby generating three parameter maps.

Two readers independently performed whole-tumor VOI delineation on SSEPI-DWI images (b = 1000 s/mm²), using dynamic contrast-enhanced and T2-weighted imaging sequences as references. They manually outlined the volume of interest (VOI) layer by layer and saved these delineations. The software then automatically transferred these VOIs to each parameter map, yielding the calculation results. For the SMS-SSEPI-DWI sequence measurements, the same VOIs saved from SSEPI-DWI were used as the regions of interest, and the results were recorded and calculated. Each reader performed two measurements and averaged the lesion values. The final value for each parameter was derived from the averaged values of both readers.

The two readers independently assessed image artifacts, imaging sharpness, lesion conspicuity, and overall image quality for all images from the two sets of DWI sequences; they also assessed FROC-DWI-derived parameter maps (D map, β map, and µ map) and ADC maps using a 5-point Likert scale. Image artifacts were regarded as motion artifacts, susceptibility artifacts, and geometric distortions (1 = severe, 2 = moderate, 3 = mild, 4 = minimal, 5 = none). Imaging sharpness was determined according to the appearance of breast tissue edges (1 = severe blurring, 2 = moderate blurring, 3 = mild blurring, 4 = minimal blurring, 5 = sharp and no blurring). Lesion conspicuity was assessed based on the contrast between suspicious lesions and surrounding background tissue (1 = none, 2 = minimal, 3 = mild, 4 = moderate, 5 = severe). Overall image quality comprised a comprehensive consideration of image artifacts, imaging sharpness, and lesion conspicuity (1 = insufficient diagnostic, 2 = poor and definitely affecting interpretation, 3 = moderate and potentially affecting interpretation, 4 = good and not affecting interpretation, 5 = excellent) [32]. The average values assessed by the two readers were taken as the final result.

Additionally, regions of interest (ROI) were plotted where the lesions appeared largest in the SSEPI-DWI and SMS-SSEPI-DWI sequences, avoiding blood vessels and necrosis zone. The SNR and CNR for each b-value image in both sequences were calculated separately. The SNR was defined as the ratio of the mean signal intensity ($\:{S}_{lesion}$) of the lesion ROI to the standard deviation of the air background ($\:{\sigma\:}_{Background}$) [33]

$$\:SNR={S}_{lesion}/{\sigma\:}_{Background}$$

The following formula was used to calculate CNR:

$$\:CNR=\frac{{S}_{lesion}-{S}_{tissue}}{\sqrt{{{\sigma\:}_{lesion}}^{2}+{{\sigma\:}_{tissue}}^{2}}}$$

$\:{\:\:\:\:\:\:\:\:\:S}_{lesion}$: mean signal intensity of lesion ROI, $\:{S}_{tissue}$: mean signal intensity of normal breast tissue, $\:{\sigma\:}_{lesion}$ and $\:{\sigma\:}_{tissue}$: standard deviations of lesion ROI and normal breast tissue, respectively [34].

### Statistical analysis

Statistical analyses were conducted using SPSS (version 23.0; SPSS, Inc., Chicago, IL, USA), MedCalc (version 20.0; MedCalc Software Ltd., Ostend, Belgium), and R (version 4.0.0; http://www.r-project.org/) softwares. Intraclass correlation coefficient (ICC) values, utilized to assess Intra- and inter-reader agreement, were categorized as follows: ICC ≤ 0.20, poor agreement; 0.21–0.40, fair agreement; 0.41–0.60, moderate agreement; 0.61–0.80, good agreement; and 0.81-1.00, excellent agreement [35]. Normality assessments were performed using the Kolmogorov-Smirnov test for all quantitative parameters. Comparative analyses of quantitative clinical data, FROC-DWI-derived parameters, and image quality scores were conducted using either independent-samples *t*-tests or the Mann-Whitney *U* test. For data that conform to a normal distribution, an independent samples *t*-test was applied for analysis. For data that do not conform to a normal distribution, a Mann-Whitney *U* test was used for analysis. The results were expressed as means ± standard deviations. Correlations between the two sets of DWI-derived parameters were evaluated by Spearman correlation analysis, with correlation coefficients (*r*) categorized as follows: *r* ≤ 0.24, little or no correlation; 0.25–0.49, fair correlation; 0.50–0.74, moderate correlation; and 0.75-1.00, good correlation [32]. Consistency between the two groups of FROC-DWI-derived parameters was assessed using Bland-Altman plots. Multivariate logistic regression was conducted to analyze FROC-DWI-derived quantitative parameters from both sets, thereby establishing a prediction model for distinguishing between benign and malignant breast lesions. Nomogram plots were generated based on the results of multivariate logistic regression, and optimal cutoff values were selected using the Youden index. The Delong test was utilized to identify significant differences in the area under the curve (AUC) of each receiver operating characteristic (ROC) curve [36]. Calibration with bootstrapped resampling was used to reduce the overfitting bias. Additionally, the Hosmer-Lemeshow goodness-of-fit test was performed to compare the predicted and actual response probabilities of the nomogram. The decision curve analysis (DCA) was also performed to quantify their clinical net benefits. The threshold for statistical significance was set to *P* < 0.05.

## Results

### Patients and lesions

Among the 178 patients, 73 had benign breast lesions: 45 fibroadenomas, 17 breast adenopathies, 8 intraductal papillomas, and 3 lobulated tumors. The remaining 105 patients had malignant breast lesions: 95 invasive carcinomas, 3 mucinous carcinomas, and 7 papillary carcinomas. The mean diameters of benign and malignant lesions did not significantly differ (mean ± standard deviation: 13.22 ± 10.31 mm vs. 18.01 ± 12.15 mm, respectively; *P* = 0.253).

### Intra- and inter-reader agreement

Analysis of consistency in the overall evaluation of DWI sequence image quality by each reader revealed excellent agreement: Reader 1 exhibited ICC values of 0.868 to 0.951 for SSEPI-DWI and 0.829 to 0.945 for SMS-SSEPI-DWI. Reader 2 showed ICC values of 0.872 to 0.955 for SSEPI-DWI and 0.834 to 0.938 for SMS-SSEPI-DWI. The overall assessment of image quality for SSEPI-DWI and SMS-SSEPI-DWI sequences between the two readers revealed ICC values of 0.812 to 0.940 and 0.836 to 0.938, respectively (Table 2), indicating consistently excellent agreement.

**Table 2:** Quality features and intraclass correlation coefficients of SSEPI-DWI and SMS-SSEPI-DWI sequences

	Reader 1			Reader 2			ICC	
	SSEPI-DWI (95% CI)	SMS-SSEPI-DWI(95% CI)	*P*	SSEPI-DWI (95% CI)	SMS-SSEPI-DWI(95% CI)	*P*	SSEPI-DWI (95% CI)	SMS-SSEPI-DWI(95% CI)
Artifacts	4.25 ± 0.51 (4.02-4.53)	4.13 ± 0.65 (3.95-4.38)	0.083	4.20 ± 0.42 (4.03-4.49)	4.18 ± 0.81 (3.83-4.39)	0.921	0.895 (0.861-0.920)	0.892 (0.859-0.915)
Image sharpness	4.03 ± 0.36 (3.82-4.28)	3.93 ± 0.28 (3.73-4.20)	0.172	4.01 ± 0.51 (3.79-4.23)	3.95 ± 0.37 (3.74-4.25)	0.532	0.940 (0.921-0.952)	0.938 (0.919-0.949)
Lesion conspicuity	4.31 ± 0.43 (4.17-4.56)	4.38 ± 0.35 (4.19-4.49)	0.618	4.35 ± 0.38 (4.20-4.57)	4.40 ± 0.19 (4.23-4.51)	0.581	0.928 (0.901-0.935)	0.935 (0.9121-0.947)
Overall image quality	4.29 ± 0.51 (4.05-4.41)	4.14 ± 0.45 (3.97-4.29)	0.079	4.32 ± 0.47 (4.13-4.48)	4.23 ± 0.63 (4.02-4.41)	0.752	0.912 (0.893-0.928)	0.863 (0.821-0.882)

Inter-reader agreement results for the SSEPI-DWI sequence were excellent across D values (ICC 0.912, 95% confidence interval [CI] 0.889–0.938), $\:\beta\:$ values (ICC 0.928, 95% CI 0.902–0.941), $\:\mu\:$ values (ICC 0.915, 95% CI 0.891–0.937), and ADCs (ICC 0.932, 95% CI 0.910–0.952). Similarly, inter-reader agreement results for the SMS-SSEPI-DWI sequence were excellent across D values (ICC 0.917, 95% CI 0.901–0.938), $\:\beta\:$ values (ICC 0.925, 95% CI 0.908–0.941), $\:\mu\:$ values (ICC 0.920, 95% CI 0.902–0.939), and ADCs (ICC 0.935, 95% CI 0.910–0.957).

### Comparison of image quality

**Fig. 1:**
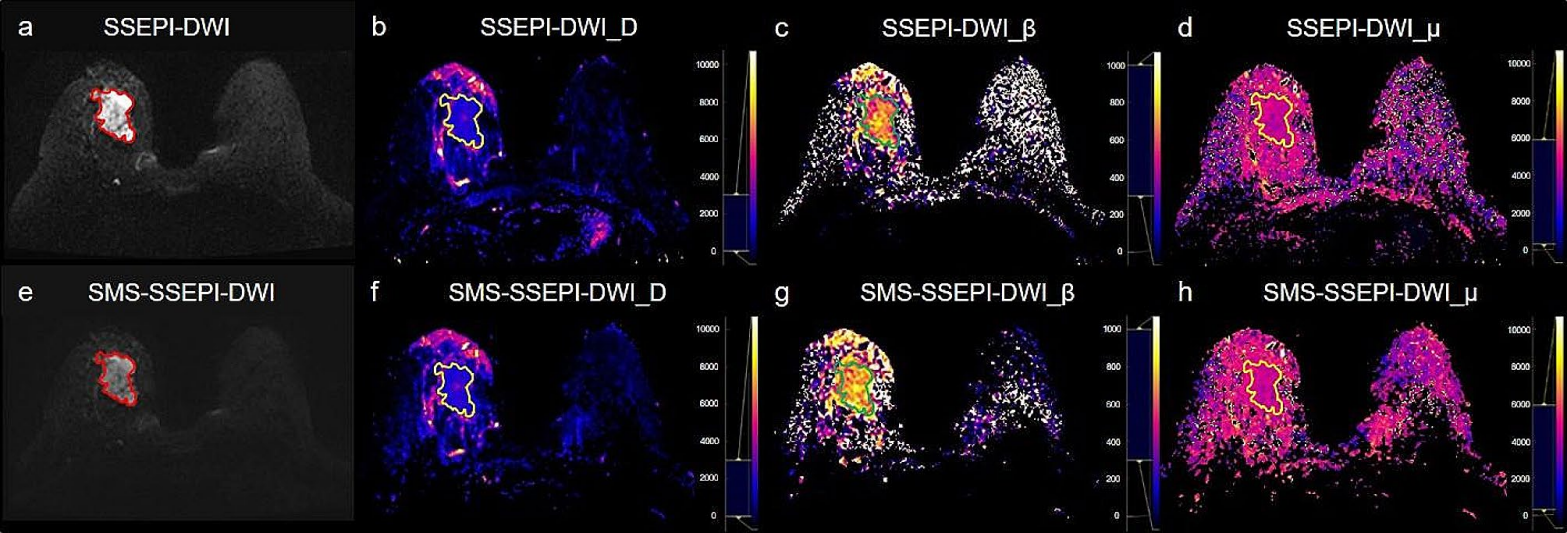
Invasive ductal carcinoma of the right breast in a 54-year-old woman. The solid line area indicates the extent of the lesion. **a**-**d**: Conventional SSEPI-DWI image (b = 1000 s/mm²) (a) and quantitative parametric maps (**b**-**d**), the SNR and CNR of the lesion area are 64.72 and 8.95 respectively (**a**), the average values of the parameters within the lesion area are D = 0.85 μm²/ms, β = 0.73, µ = 3.5 μm. **e**-**h**: SMS-SSEPI-DWI image (b = 1000 s/mm²) (**e**) and quantitative parametric maps (**f**-**h**), the SNR and CNR of the lesion area are 58.25 and 8.13 respectively (**e**), the average values of the parameters within the lesion area are D = 0.87 μm²/ms, β = 0.76, µ = 3.6 μm

Tables 2 and 3 shows the results of qualitative and quantitative comparisons of DWI sequence image quality between the two groups. The analysis revealed no significant differences between the DWI sequence groups concerning image artifacts, image sharpness, lesion conspicuity, overall image quality (all *P* > 0.05) (Table 2), as indicated in Fig. 1. Table 3 indicates that the SNR for all b-values in the SSEPI-DWI sequence are higher than those in the SMS-SSEPI-DWI sequence. However, except for the SNR on b = 2000 and 2500 s/mm², which show a statistically significant difference between the two sequences, there is no statistical difference in SNR for other b-values. In terms of CNR, there is a statistically significant difference between the two sequences on b = 2000, 2500, and 3000 s/mm², with no statistical difference in CNR observed for other images.

**Table 3:** Compare the SNR and CNR at different b-values between the SSEPI-DWI and SMS-SSEPI-DWI sequences

b (s/mm^2^)	SNR			CNR		
	SSEPI-DWI	SMS-SSEPI-DWI	*P*	SSEPI-DWI	SMS-SSEPI-DWI	*P*
0	76.15 ± 12.12	75.11 ± 20.01	0.242	12.04 ± 2.51	13.38 ± 3.62	0.732
50	75.85 ± 13.57	73.81 ± 21.73	0.531	12.63 ± 2.24	12.57 ± 3.27	0.583
80	75.29 ± 15.67	71.72 ± 25.01	0.844	11.85 ± 3.04	12.07 ± 3.16	0.615
100	74.75 ± 11.93	69.36 ± 15.05	0.782	11.32 ± 2.67	11.51 ± 3.82	0.935
150	74.35 ± 11.12	66.15 ± 20.28	0.720	10.93 ± 3.53	10.74 ± 3.48	0.786
200	73.02 ± 13.71	68.76 ± 18.62	0.377	11.15 ± 2.87	10.67 ± 2.64	0.591
400	70.85 ± 12.61	62.13 ± 20.02	0.821	10.31 ± 3.06	10.26 ± 3.70	0.558
600	67.73 ± 11.07	60.17 ± 18.52	0.518	9.63 ± 3.58	9.86 ± 2.76	0.812
800	65.97 ± 12.51	58.84 ± 17.94	0.254	9.33 ± 2.56	8.96 ± 3.22	0.462
1000	65.85 ± 13.72	58.06 ± 14.83	0.114	8.93 ± 3.48	8.86 ± 4.54	0.685
1500	63.21 ± 11.15	54.32 ± 22.71	0.061	7.53 ± 3.27	7.16 ± 2.75	0.244
2000	62.93 ± 15.76	50.75 ± 17.05	0.025	6.83 ± 3.12	5.92 ± 4.54	0.044
2500	58.39 ± 13.78	46.63 ± 20.82	< 0.001	6.14 ± 2.68	5.06 ± 3.29	0.012
3000	52.65 ± 17.07	45.52 ± 15.92	0.726	5.53 ± 3.02	4.42 ± 3.62	0.040

### Comparison of FROC-DWI parameters and ADCs

**Fig. 2:**
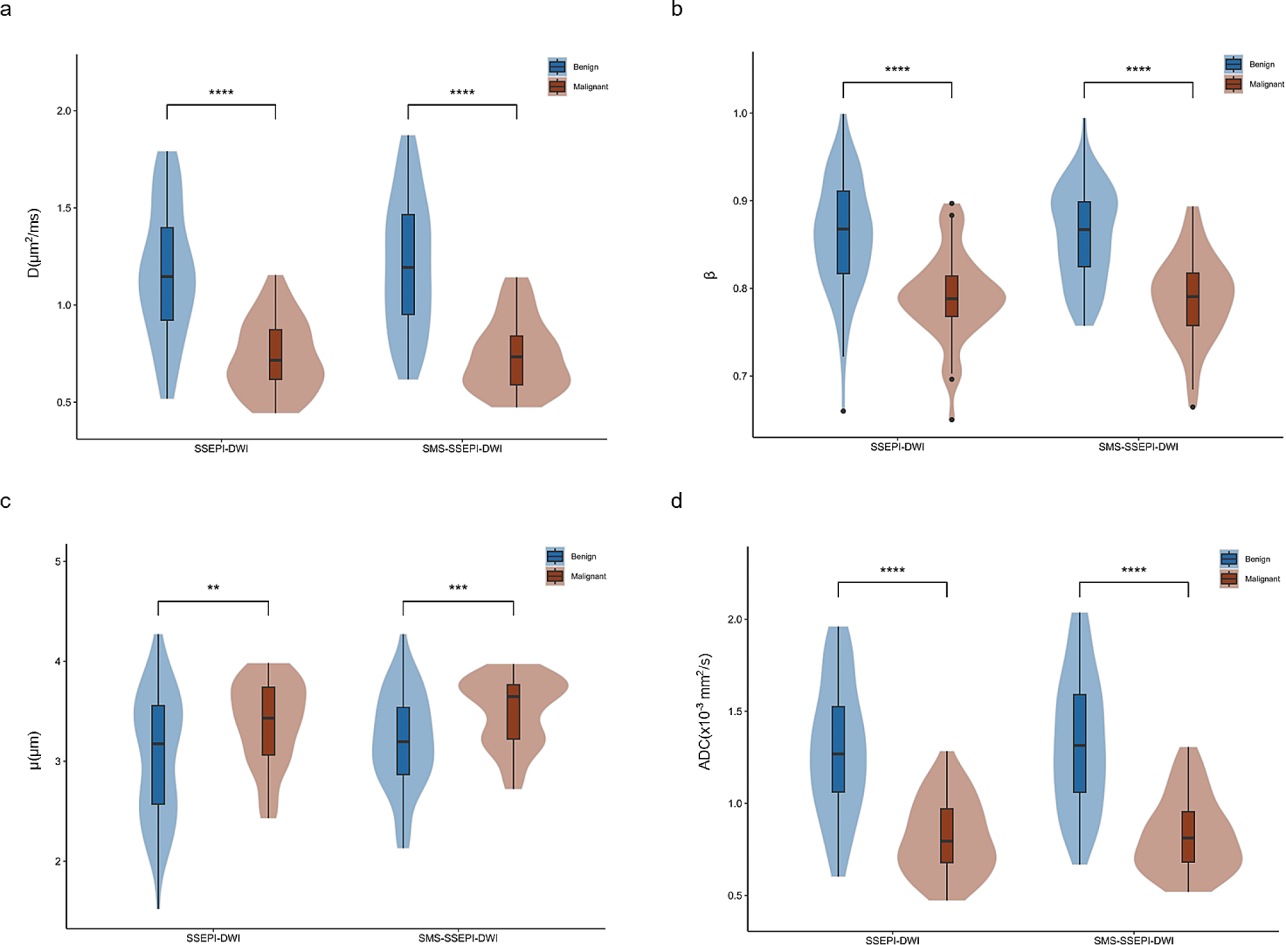
Boxplots of D values (**a**), $\:\beta\:$ values (**b**), $\:\mu\:$ values (**c**), and ADCs (**d**). **p < 0.01, ***p < 0.001,****p < 0.0001

Table 4 and Fig. 2 illustrate the mean D values, $\:\beta\:$ values, $\:\mu\:$ values, and ADCs obtained from the two groups of FROC-DWI sequences. The D values, $\:\beta\:$ values, and ADCs were significantly lower for malignant lesions than for benign lesions (all *P* < 0.001). Conversely, $\:\mu\:$ values were significantly higher for malignant lesions than for benign lesions (*P* < 0.01). Furthermore, the parameter µ exhibits a clear bimodal distribution in SMS-SSEPI-DWI (Fig. 2).

**Table 4:** DWI parameters for benign and malignant breast lesions in SSEPI-DWI and SMS-SSEPI-DWI sequences

	Benign lesion (*N* = 73)	Malignant lesion (*N* = 105)	*P*
D ($\:\mu\:$m^2^/ms) SSEPI-DWI SMS-SSEPI-DWI	1.17 ± 0.34 1.21 ± 0.34	0.73 ± 0.18 0.74 ± 0.18	< 0.001 < 0.001
$\:\beta\:$ SSEPI-DWI SMS-SSEPI-DWI	0.86 ± 0.06 0.86 ± 0.05	0.77 ± 0.06 0.79 ± 0.05	< 0.001 < 0.001
$\:\mu\:$ (µm) SSEPI-DWI SMS-SSEPI-DWI	3.06 ± 0.63 3.20 ± 0.48	3.39 ± 0.42 3.50 ± 0.34	0.006 < 0.001
ADC (×10^− 3^ mm^2^/s) SSEPI-DWI SMS-SSEPI-DWI	1.30 ± 0.36 1.33 ± 0.36	0.82 ± 0.20 0.83 ± 0.20	< 0.001 < 0.001

**Fig. 3:**
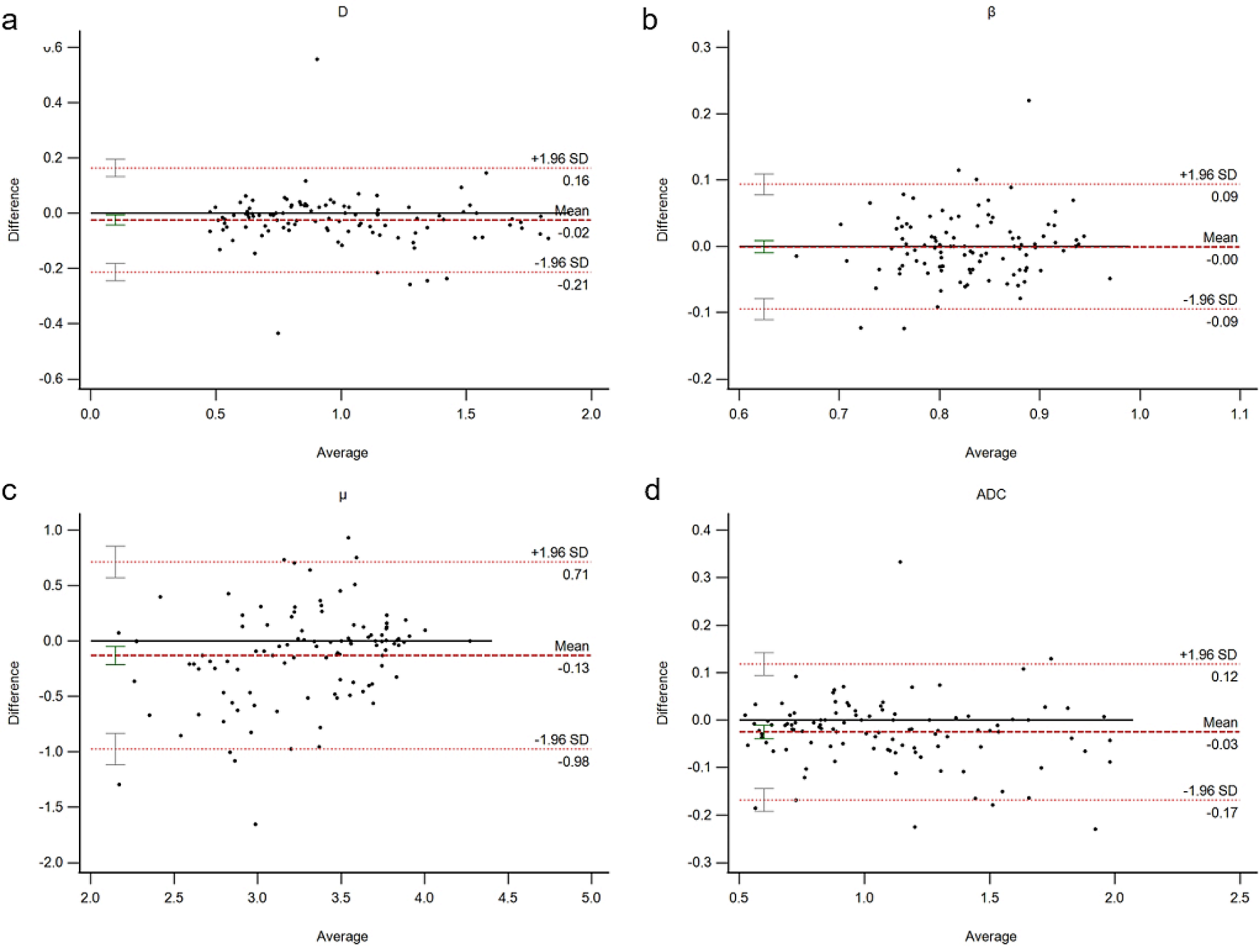
Bland-Altman plots showing minimal bias (thick red dotted line) for D values (**a**), $\:\beta\:$ values (**b**), $\:\mu\:$ values (**c**), and ADCs (**d**) between SSEPI-DWI and SMS-SSEPI-DWI sequences. The black horizontal line denotes zero difference. Thin red dotted lines indicate upper and lower 95% limits of agreement

Spearman’s correlation coefficient analyses revealed robust linear correlations for D values (*r* = 0.956, 95% CI 0.931–0.975) and ADCs (*r* = 0.983, 95% CI 0.963–0.998) between SSEPI-DWI and SMS-SSEPI-DWI sequences. However, $\:\beta\:$ values (*r* = 0.732, 95% CI 0.701–0.762) and $\:\mu\:$ values (*r* = 0.640, 95% CI 0.621–0.668) exhibited moderate correlation between the two sets of sequences (all *P* < 0.001). Furthermore, a robust linear correlation was observed between D values and ADCs from both sequence sets (*r* > 0.967, *P* < 0.001). The Bland-Altman plot (Fig. 3) depicts the mean difference values and 95% limits of agreement between the two groups of FROC-DWI sequence-derived parameters and ADCs: D values (-0.02, -0.21 to 0.16), $\:\beta\:$ values (-0.00, -0.09 to 0.09), $\:\mu\:$ values (-0.13, -0.98 to 0.71), and ADCs (-0.03, -0.17 to 0.12).

### Diagnostic performance

**Fig. 4:**
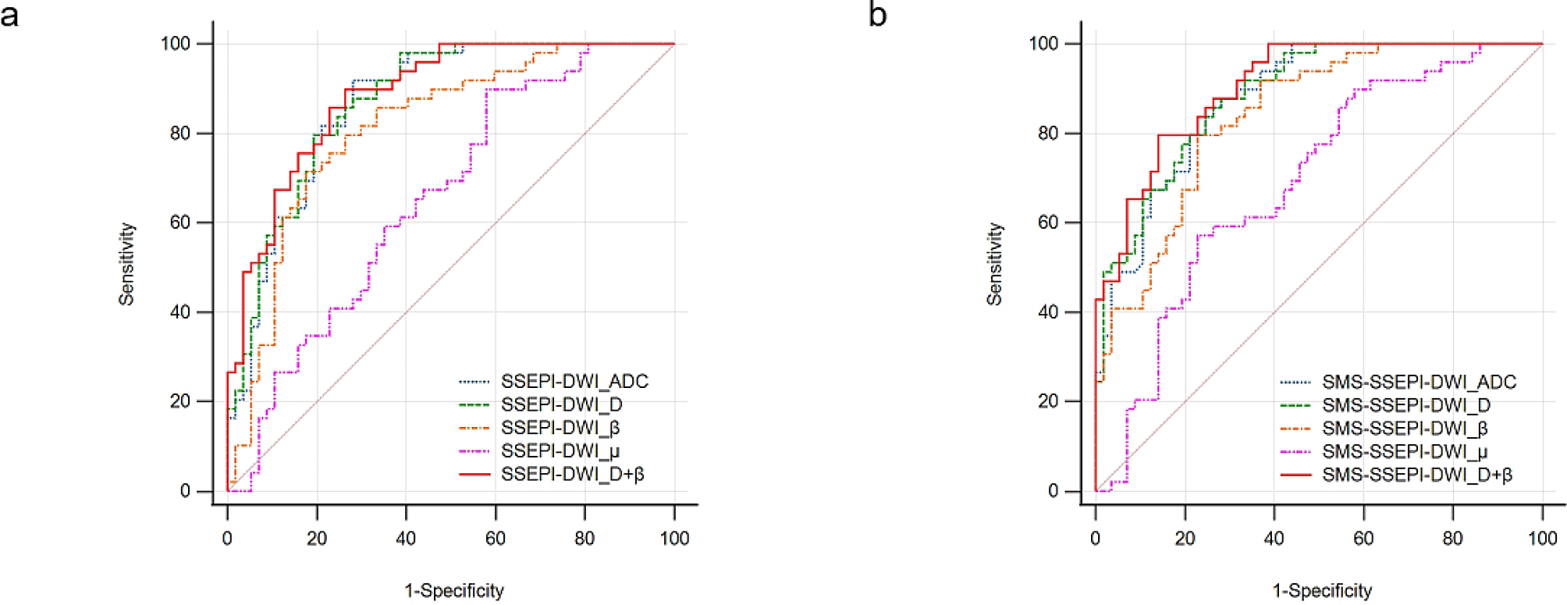
Receiver operating characteristic curves (ROC) for ADCs; the FROC-DWI-derived parameters D, $\:\beta\:$, and $\:\mu\:$; and the combination of D and $\:\beta\:$ for SSEPI-DWI (**a**) and SMS-SSEPI-DWI (**b**) sequences

Table 5 and Fig. 4a and b show the ROC analysis results for derived parameters (D, $\:\beta\:$, and $\:\mu\:$ values) and ADCs in both groups of FROC-DWI sequences. In the single-parameter analyses of SSEPI-DWI and SMS-SSEPI-DWI, the D value demonstrated the highest accuracy (83.52%, 81.88%), largest AUC (0.88, 0.89), and highest sensitivity (91.84%, 87.77%). $\:\beta\:$ displayed the highest specificity (82.46%, 77.37%).

**Table 5:** ROC analysis results for SSEPI-DWI and SMS-SSEPI-DWI sequences

	Cutoff	AUC (95% CI)	Sensitivity (%)	Specificity (%)	Accuracy (%)
D SSEPI-DWI SMS-SSEPI-DWI	0.97 0.92	0.88 (0.79–0.93) 0.89 (0.81–0.95)	91.84 87.77	71.93 71.91	83.52 81.88
$\:\beta\:$ SSEPI-DWI SMS-SSEPI-DWI	0.82 0.82	0.81 (0.73–0.88) 0.84 (0.76–0.91)	71.43 79.59	82.46 77.37	76.53 78.03
$\:\mu\:$ SSEPI-DWI SMS-SSEPI-DWI	3.09 3.38	0.66 (0.56–0.75) 0.69 (0.60–0.78)	89.80 57.14	42.11 77.19	64.82 65.84
ADC SSEPI-DWI SMS-SSEPI-DWI	0.99 1.07	0.87 (0.79–0.93) 0.88 (0.80–0.94)	90.59 87.76	72.70 71.93	80.15 80.02
D + β SSEPI-DWI SMS-SSEPI-DWI	0.35 0.64	0.91 (0.83–0.95) 0.92 (0.85–0.96)	90.80 89.59	75.68 75.96	85.55 83.17

**Fig. 5:**
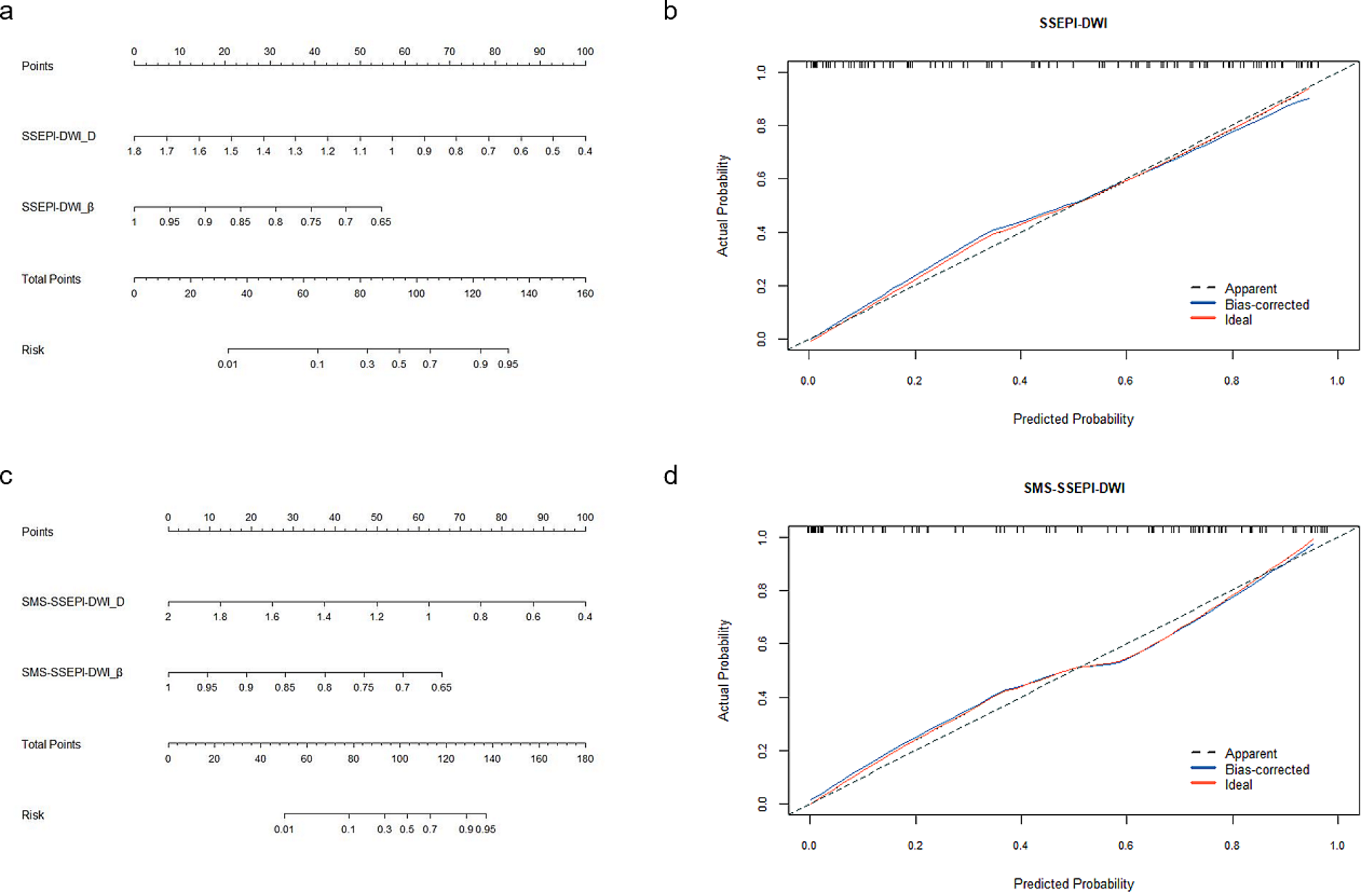
Nomograms for predicting malignant breast lesions with SSEPI-DWI (**a**) and SMS-SSEPI-DWI (**c**) sequences. Calibration plots for internal validation of malignant breast lesions with SSEPI-DWI (**b**) and SMS-SSEPI-DWI (**d**) sequences. The dotted line represents the predictive calibration curve of the nomogram; the orange solid line represents the ideal standard curve. The dotted line demonstrates a close fit to the orange ideal dotted line, indicating that the nomogram exhibits better predictive accuracy

**Fig. 6:**
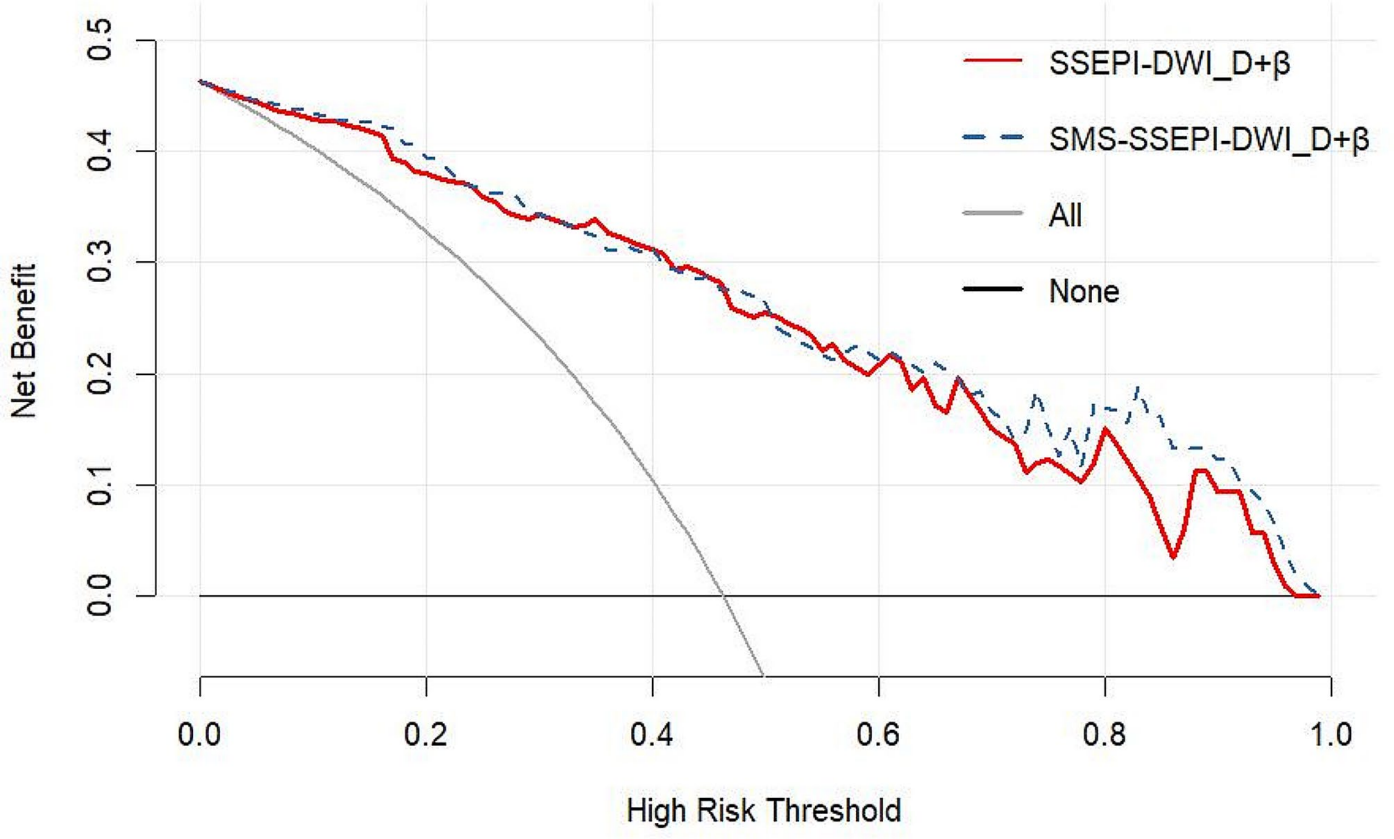
Decision curve analysis(DCA) of the prediction model. The black horizontal solid line indicates that all lesions are benign. The solid gray oblique line indicates that all lesions are malignant. The red solid line is the decision curve of the prediction model constructed by SSEPI-DWI, and the blue dashed line is the decision curve of the prediction model constructed by SMS-SSEPI-DWI.

Multivariate logistic regression was used to construct a prediction model with FROC-DWI parameters (D, $\:\beta\:$, and $\:\mu\:$ values). The results showed that D and $\:\beta\:$ values were significant in both sequences (*P* = 0.001 and *P* = 0.035, respectively), whereas $\:\mu\:$ values were not (*P* = 0.095), leading to exclusion of $\:\mu\:$ values from the model. In SSEPI-DWI and SMS-SSEPI-DWI, D+$\:\beta\:$ demonstrated the highest AUC (0.91, 95% CI 0.83–0.95; 0.92, 95% CI 0.85–0.96) and accuracy (85.55%, 83.17%) in terms of diagnosing benign and malignant breast lesions (Table 5; Fig. 4a and b). The Delong test revealed significant differences in AUCs for D+$\:\beta\:$ values, D values, $\:\beta\:$ values, and ADCs between the two groups (*P* < 0.05). However, there were no significant differences in AUCs for D+$\:\beta\:$ values, D values, $\:\beta\:$ values, and ADCs within the groups (*P* > 0.05). Nomogram plots from both DWI sequences are presented in Fig. 5a and c. The Hosmer-Lemeshow test revealed that the prediction models exhibited good fit for both SSEPI-DWI (*P* = 0.546) and SMS-SSEPI-DWI (*P* = 0.481) sequences (Fig. 5b and d). Additionally, decision curve analysis(DCA) (Fig. 6) demonstrated that the prediction models from both groups yielded results within the probability threshold, supporting clinical decision-making.

## Discussion

The results of this study highlight the potential for FROC-DWI model parameters (D, $\:\beta\:$, and $\:\mu\:$ values) and ADC to distinguish benign and malignant breast lesions on SMS-SSEPI-DWI and SSEPI-DWI. The model comparison showed that D values had the highest diagnostic accuracy, sensitivity, and AUC, superior to ADC, whereas $\:\beta\:$ values demonstrated higher sensitivity compared with ADC. Notably, SMS-SSEPI demonstrated the highest AUC (89%) for D values among all quantitative parameters. The image quality and FROC-derived parameters of SMS-SSEPI-DWI and SSEPI-DWI sequences displayed high consistency and reproducibility in differential diagnoses of benign and malignant breast lesions, while substantially reducing scan time (by 49.8%).

The present study showed that D values both from SSEPI-DWI and SMS-SSEPI-DWI were lower for malignant lesions than for benign lesions, and had the higher diagnostic efficiency, consistent with previous FROC model studies regarding other tumors [11, 12, 16, 20]. Similar to the diffusion coefficients in other diffusion models (IVIM, DKI, and CTRW), the parameter D in the FROC-DWI model is influenced by tumor cell density, the integrity of cell membranes, and other structural properties that affect the diffusion process of water in tissues [9]. This may be related to the increase in cellular density and the reduction of extracellular space distortion that occurs during the development of breast cancer [3, 7]. Although the single-exponential DWI model is widely used due to its short scan time and simple post-processing, it has many limitations [3]. The ADC values are influenced by cellular structural changes and cannot fully characterize the non-Gaussian diffusion within heterogeneous tumor environments. D values, calculated using numerous increasing b-values in the FROC-DWI model, more accurately reflect actual diffusion processes by considering the non-Gaussian distribution effect of tumor heterogeneity.

β values, a new type of FROC-DWI parameter, are negatively correlated with tissue microstructure heterogeneity and complexity [10]. This is an important tissue characteristic. In our study, the β values in the two sets of FROC-DWI sequences for breast cancer were significantly lower than those for benign breast lesions. This may be attributed to the irregular cell morphology, increased vascular density in breast cancer, and the presence of different subpopulations of tissue cells, resulting in greater structural heterogeneity. This finding is also consistent with other studies on benign and malignant lesions using FROC-DWI in different regions [11, 16, 20]. He et al. [3], in their study using the IVIM and DKI models for breast lesions, found that the kurtosis-based diffusion heterogeneity index MK was significantly higher in malignant lesions compared to benign ones (MK being positively correlated with tissue microstructure). Additionally, several studies on breast lesions using the CTRW model have shown results consistent with those from FROC-DWI, with the β values in malignant lesions being significantly lower than in benign lesions [8, 37].

The µ from FROC-DWI parameter is negatively correlated with the average diffusion length of water molecules [9]. In our study of the two DWI sequences, the µ values in malignant breast lesions were higher than those in benign lesions, which may reflect the significant cell proliferation in malignant breast tumors, restricted diffusion of water molecules, and an average diffusion length shorter than that in benign lesions, leading to an increase in µ values. However, in our study, the µ values in malignant breast lesions showed a clear bimodal distribution in SMS-SSEPI-DWI, which could potentially be a point of differentiation between benign and malignant lesions. Although our current data results cannot fully explain this phenomenon, it warrants further investigation. However, previous studies revealed conflicting results concerning µ values [11–13, 16]. In the studies by SUI et al. [12, 13] on pediatric brain tumors and adult gliomas, the µ values for high-grade brain tumors were consistently lower than those for low-grade tumors, suggesting that µ values have a role in predicting tumor grading. In the research by LUO et al. [16] on pulmonary nodules, the µ values for malignant pulmonary nodules were significantly higher than those for benign nodules. However, in studies on the prostate, there was no difference in µ values between prostate hyperplasia and prostate cancer [11]. Incorporating the FROC fitting formula and findings from previous research, it has been observed that when β approaches 1.0, the stability of the µ value becomes uncontrollable, which may also be one of the reasons for the inconsistency in µ values across studies. Therefore, the clinical research value of µ values still requires further exploration.

Nomograms are intuitive and visual tools that can facilitate clinical work. In our study, the combination of multiple parameters in the two sets of FROC-DWI models, especially the D and β values, can partially enhance the diagnostic efficacy for malignant breast lesions. The (D + β) nomogram models constructed based on SSEPI-DWI and SMS-SSEPI-DWI FROC demonstrated high AUC values (0.91, 0.92) and accuracy (85.55%, 83.17%), but their sensitivity and specificity may be hindered by the inherent structural heterogeneity of breast tissue. However, due to the limited total sample size and the number of indicators in the predictive model of this study, the extrapolation application of the nomogram model still requires more validation.

Simultaneous Multi-Slice (SMS) technology enables simultaneous excitation of multiple slices using multi-band RF pulses and reconstructs images using coil sensitivity, gradients, or RF encoding. This approach can halve scanning time or double the number of slices acquired, enhancing patient comfort and efficiency. [20, 21, 24]. Machida et al [30] found that SMS-SSEPI-DWI improved visual assessment of breast tumors in 55 cases, with consistent ADC values and reduced acquisition time compared to SSEPI-DWI [29]. Tang et al. noted similar diagnostic efficacy between SSEPI and SMS-rs EPI DWI with 8 b-values, although the TR was shortened to 2350 ms, below the ESBR’s recommendation of over 3000 ms for breast DWI sequences [29, 38]. Due to the use of 14 b-values in this study, with the highest b-value of 3000 s/mm², to balance image quality and patient tolerance, SSEPI-DWI was chosen for multi-b-value DWI research.

In the subjective evaluation of this study, SMS-SSEPI-DWI had comparable image quality to conventional SSEPI-DWI (in terms of artifacts, image sharpness, lesion conspicuity, and overall image quality), and these results are consistent with previous research conclusions [22–24]. Despite a lower SNR in SMS-SSEPI-DWI compared to SSEPI-DWI, particularly at b ≥ 2000 s/mm², the image quality remains clinically comparable. The reason may be that the SNR of SSEPI at ultra-high b-values is already low, and with the addition of SMS technology, the TR is further reduced, leading to a faster decrease in SNR and CNR. Previous studies showed that the acceleration factor of 2 in SMS-SSEPI-DWI offers a balance between reduced scanning time and acceptable image quality [22, 27]. Therefore, this study only compared the SMS-SSEPI-DWI sequence with an acceleration factor of 2. The scanning time was reduced from 7min48s (SSEPI-DWI) to 3min55s (SMS-SSEPI-DWI), a reduction of about 49.8%.

This study has some limitations. Firstly, this study is a single-center retrospective study with an uneven distribution of benign and malignant patients, and there is a possibility of bias in the selection of the patient cohort, which may introduce bias into the statistical analysis. Therefore, it is necessary to conduct studies with multiple research centers, equipment from various vendors [24], and larger sample sizes [15] to improve the research outcomes. Secondly, MRI DWI sequences were not rescanned and remeasured for the same patient. According to the recommendations of the Quantitative Imaging Biomarker Alliance (QIBA), when providing tumor imaging biomarkers, rescanning and remeasuring the sequence is preferred for the repeatability and reproducibility of MRI quantitative techniques [39, 40]. However, in actual clinical practice, there are certain limitations in terms of time, cost, patient tolerance, and compliance, making the rescanning and remeasuring of these DWI sequences difficult to achieve. Thirdly, we only assessed lesions larger than 5 mm because, for smaller lesions, the partial volume effect [41] needs to be considered when measuring FROC-DWI derived parameters and ADC values. Finally, because the FROC model examines tissue structure heterogeneity, its parameter values are closely related to histopathology. It is important to recognize that the presence of different subtypes within benign and malignant breast lesions might have influenced the results of the present study.

## Conclusion

The use of an SMS-SSEPI-DWI FROC model based on non-Gaussian distribution appears to be clinically viable for distinguishing between benign and malignant breast lesions. The derived parameters, particularly D and $\:\beta\:$ values, have superior diagnostic potential compared with ADCs. Furthermore, SMS-SSEPI-DWI can substantially reduce scan time while maintaining image quality and relevant diagnostic parameters compared with SSEPI-DWI. In the future, we plan to expand the application of SMS technology combined with the FROC-DWI model to other types of tumors.

## Data Availability

The data that support the findings of this study are available from the corresponding author but restrictions apply to the availability of these data, which were used under license for the current study, and so are not publicly available. Data are however available from the authors upon reasonable request and with permission of the corresponding author.

## Abbreviations

MRI: Magnetic resonance imaging
DWI: Diffusion-weighted imaging
ADCs: Apparent diffusion coefficients
FROC: Fractional-order calculus
SMS: Simultaneous multi-slice
SSEPI-DWI: Single-shot echo planar imaging DWI
ROI: Regions of interest
SNR: Signal-to-noise
CNR: Contrast-to-noise
ROC: Receiver operating characteristic
AUC: Under the curve
ICC: Intraclass correlation coefficient
DCA: Decision curve analysis
